# *De novo* transcriptome assembly of hyperaccumulating *Noccaea praecox* for gene discovery

**DOI:** 10.1038/s41597-023-02776-x

**Published:** 2023-12-01

**Authors:** Valentina Bočaj, Paula Pongrac, Sina Fischer, Matevž Likar

**Affiliations:** 1 University of Ljubljana, Biotechnical Faculty, Jamnikarjeva 101, SI-1000 Ljubljana, Slovenia; 2 Jožef Stefan Institute, Jamova 39, SI-1000 Ljubljana, Slovenia; 3 University of Nottingham, Future Food Beacon of Excellence and School of Biosciences, LE12 5RD Loughborough, United Kingdom

**Keywords:** Plant molecular biology, Plant genetics

## Abstract

Hyperaccumulators are a group of plant species that accumulate high concentrations of one or more metal(loid)s in their above-ground tissues without showing any signs of toxicity. Several hyperaccumulating species belong to the Brassicaceae family, among them the Cd and Zn hyperaccumulator *Noccaea praecox*. In this paper, we present *de novo* transcriptome assembled from two naturally occurring *N. praecox* populations growing in (i) metal-enriched soil and (ii) soil non-contaminated with metals (control site). Total RNA was extracted from the leaves of both populations. We obtained 801,935,101 reads, which were successfully assembled and annotated. The resulting assembly contains 135,323 transcripts, with 103,396 transcripts (76.4%) annotated with at least one function and encoding 53,142 putative proteins. Due to its close relationship with the hyperaccumulating model species *N. cearulescens*, it will be possible to derive protein functions from sequence comparisons with this species. Comparisons will highlight common and differing pathways of metal acquisition, storage, and detoxification which will allow us to expand our knowledge of these processes.

## Background & Summary

Hyperaccumulators are defined as plant species that can accumulate extraordinarily high concentrations of one or more metal(loid)s in the above-ground biomass (especially leaves) without apparent toxicity symptoms^1,2^. Concentrations of metal(loid)s in the leaves of a hyperaccumulating species could be up to 1,000-fold higher compared to non-hyperaccumulators^2,3^. To date, approximately 500 plant taxa (0.2% of all angiosperms) are acknowledged to hyperaccumulate metal(loid)s, with several belonging to the Brassicaceae family^4,5^. Although most hyperaccumulators are defined as nickel (Ni) hyperaccumulators, they accumulate other metal(loid)s as well, including arsenic (As), cadmium (Cd), cobalt (Co), chromium (Cr), copper (Cu), manganese (Mn), lead (Pb), antimony (Sb), selenium (Se), thallium (Tl) and zinc (Zn)^6–8^. Metal hyperaccumulation is of interest for several reasons, which include the biofortification of staple crops^9^, phytoremediation^10,11^, and food protection against toxic metal(loid)s^12^.

Some hyperaccumulating species in Brassicaceae were identified in the genus *Noccaea*, which includes a well-known hyperaccumulating model species, *Noccaea caerulescens*. The last hyperaccumulating representative of this genus, identified up to date, was *Noccaea praecox*, a hyperaccumulator of Cd and Zn^13^. In *N. praecox* leaves, Zn is primarily stored in the epidermis, whereas most of the Cd is distributed within the mesophyll^14^. Both metals were also found in the seeds and were preferentially localized in the epidermis of cotyledons^15^. Even though it is known Brassicaceae do not form mycorrhizal associations, it was demonstrated *N. praecox* forms symbiosis with arbuscular mycorrhizal fungi, which improved the plant’s nutrient uptake^13,16^.

Although *N. praecox* is a well-characterized hyperaccumulating species, in contrast to its closely related *N. caerulescens* and *N. goesingense* no studies were performed on the transcriptome or genome of *N. praecox*^17,18^. Despite extensive genomic data acquisition in recent years, current knowledge of gene networks in hyperaccumulators providing physiological responses to environmental changes remains incomplete. As such, RNA-seq of a new hyperaccumulating *Noccaea* species and validation of metabolic pathways and regulation cascades observed in the model species *N. caerulescens* could facilitate physiological and molecular studies of these species.

Here we provide the transcriptome of *N. praecox*. To capture the expression of genes relevant to metal homeostasis under high and low metal load, we analyzed samples from two localities (metal-enriched and non-polluted soils). Detailed accumulation data is available for these sites^17^. A transcriptome comparison between the two populations and analysis of differentially expressed genes with subsequent models on potential detoxification pathways will be the object of future studies.

## Methods

### Sample collection

Samples representing the whole flowering plant, including the rhizosphere and bulk soil, were collected in Spring 2022 in Lokovec (N 46° 2′ 39.2706″, E 13° 46′ 8.9934″) and Žerjav (N 46° 28′ 26.1258″, E 14° 51′ 56.0118″) and transferred to the lab. Soil from Lokovec is not contaminated soil, whereas Žerjav is metal-contaminated due to the past mining and smelting activities in the region. Leaves of four plants of *N. praecox* from each site were sampled, flush-frozen in liquid nitrogen, and stored at −80 °C until further analysis.

### Total RNA extraction

Total RNA from plant leaves of *N. praecox* from both sites was extracted according to the protocol for RNA extraction from plant tissues^19^. Frozen leaves were ground and homogenized in 400 μL of Z6-buffer containing 8 M guanidinium-HCl, 20 mM MES, and 20 mM EDTA (pH = 7). After the addition of 400 μL of phenol:chloroform:isoamyl alcohol (25:24:1), samples were vortexed and centrifuged for phase separation for 10 minutes at 20,000 g. The upper aqueous phase was transferred to a new microcentrifugation tube, and 0.05 volumes of 1 N acetic acid and 0.7 volumes of 96% ethanol were added. After overnight precipitation at −20 °C, samples were centrifuged for 20 min at 4 °C (20,000 g). The pellet was washed with 200 μL sodium acetate (pH = 5.2) and 70% ethanol. After drying, RNA was dissolved in 30 μL of ultrapure water. The removal of the DNA in the RNA samples was carried out using RNAse free DNAse according to the protocol of RevertAid First Strand cDNA Synthesis Kit (Thermo Scientific). Samples were then stored at −80 °C until further analysis.

### Quality control of total RNA, library preparation, and sequencing

RNA quality checks, library preparation, and sequencing were performed by Macrogen company. RNA Integrity Number (RIN) was calculated using Agilent Technologies 2100 Bioanalyzer. Three samples per population (six altogether) were used for further analysis. The cDNA libraries of six samples of *N. praecox* from both locations were constructed following the manufacturer’s instructions using the TruSeq Stranded mRNA Sample Preparation Kit (Illumina, San Diego, USA). All cDNA libraries were sequenced on the Illumina NovaSeq. 6000 platform using 2 × 150 PE (paired-end sequencing with 150 nt reads). Corresponding read depths are presented in Table 1.

**Table 1:** Description of samples used to acquire RNA-seq data, with the number of reads retained or removed at various stages of preprocessing.

Sample ID	Habitat	Biological Replicate	Raw Reads	After kmer correction	The final number of reads	Retained reads [%]
Np_Lo_13	control	1	147,650,836	129,641,042	122,012,486	82.6
Np_Lo_14	control	2	121,309,051	106,229,479	105,798,037	87.2
Np_Lo_15	control	3	132,859,384	115,160,311	114,498,350	86.2
Np_Ze_12	metal-enriched	1	140,954,632	122,472,560	122,138,223	86.7
Np_Ze_14	metal-enriched	2	141,952,259	124,684,580	124,450,757	87.7
Np_Ze_15	metal-enriched	3	117,208,939	102,304,432	100,420,524	85.7
Total			801,935,101	700,492,404	689,318,377	86.0

Leaves of *Noccaea praecox* (Np) were collected either on the control site (Lo) or metal-enriched site (Ze).

### *De novo* transcriptome assembly

**Fig. 1:**
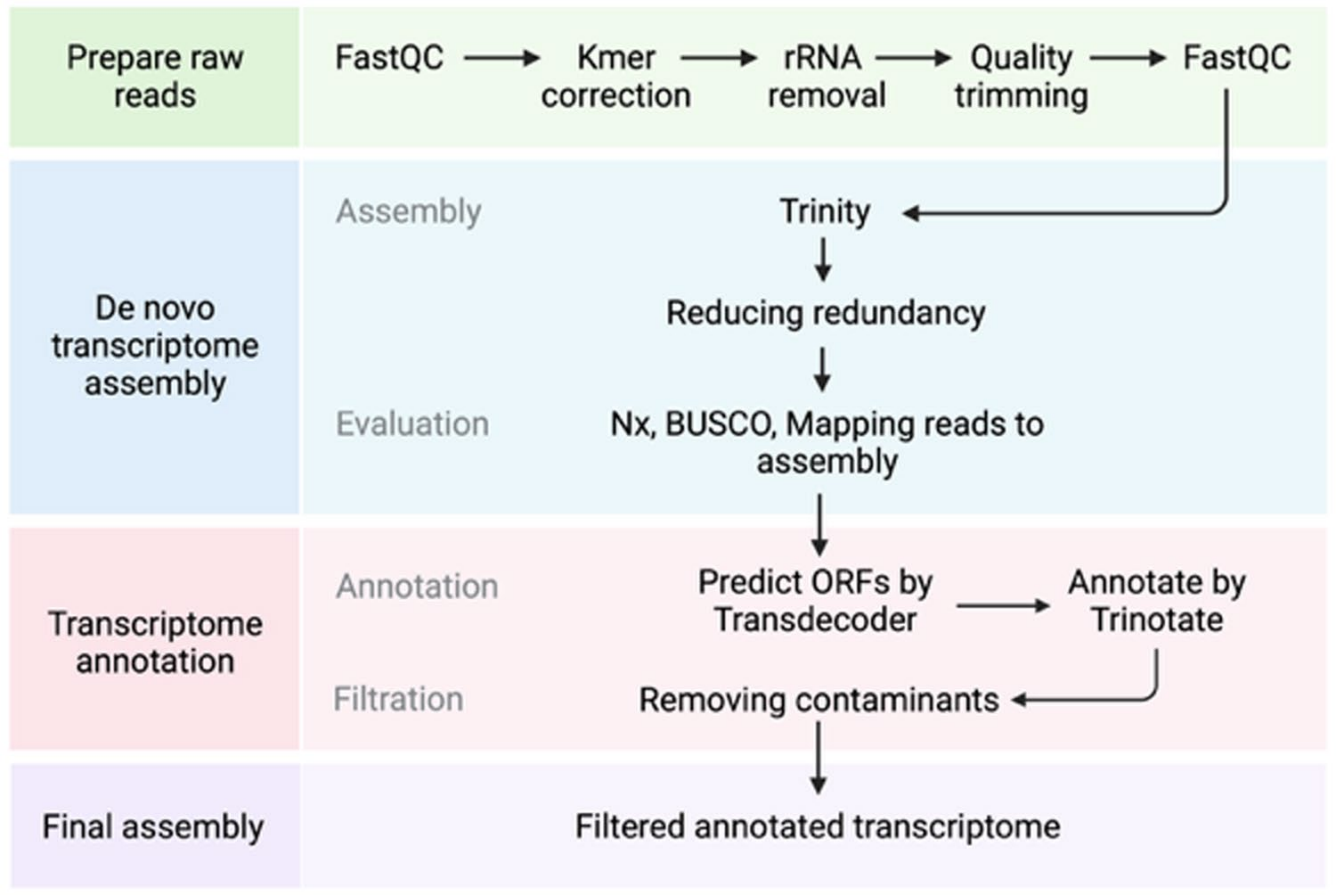
Flowchart of the bioinformatic analysis pipeline.

The overall bioinformatic workflow of transcriptome assembly and annotation is summarized in Fig. 1. We used six biological samples, three from contaminated soil and three from non-contaminated soil for the assembly. Raw reads were processed with RCorrector v1.0.5^20^ installed through Anaconda. Uncorrectable reads were removed using FilterUncorrectabledPEfastq.py python script [https://github.com/harvardinformatics/TranscriptomeAssemblyTools]. Cleaned reads were further processed for adapter removal and quality trimming using TrimGalore v0.6.2 installed through Anaconda with default parameters and–length 50 -q 5–stringency 1 -e 0.1. Ribosomal RNAs potentially still present after polyA capture were removed through alignment against the SILVA Ribosomal database (Release 138) with Bowtie2 v2.5.1^21^. Read quality was assessed before and after the processing of reads with FastQC v0.11.8^22^. Retained reads were assembled with Trinity v2.13.2^23^ using default options and --SS_lib_type RF and --min_contig_length 300 (minimum length of contigs 300 nt). Assembly retained the sample information and allows differential expression analysis using native Trinity scripts and deposited raw reads (see Data Records). Finally, we used CD-HIT-EST v4.8.1^24^ to reduce transcript redundancy with the following options: -c 0.90 -n 9 -d 0 -M 0 -T 30 -s 0.9 -aS 0.9. The resulting unique genes (unigenes) were used for the quality check of the assembly and annotations. To find contigs originating outside of the *N. praecox* transcriptome, we used the NCBI Foreign Contamination Screen (FCS) caller (https://github.com/ncbi/fcs), which flagged 39,641 sequences for removal.

The quality of the assembly was first analyzed with TrinityStats.pl, and the final transcriptome completeness was estimated using the Benchmarking Universal Single-Copy Orthologs (BUSCO) v5^25^ against the conserved single-copy Viridiplantae genes database on the server gVolante [https://gvolante.riken.jp] Finally, filtered reads were mapped back to the transcriptome to evaluate individual mapping rate with Bowtie2 and ExN50 was generated by Trinity accessory scripts.

### Differentially expressed genes

The original sequence reads were mapped to the assembly using the Kallisto pseudoaligner^26^ and differentially expressed genes (DEGs) were defined as genes having a false discovery rate (FDR) ≤0.05 and an absolute log_2_ fold change value ≥1 in R using DESeq2 v1.40.2 library^27^.

### Transcriptome annotation

Transcriptome assembly annotation was performed using Trinotate v.4.0.0 pipeline [http://trinotate.github.io]. First, contigs were scanned with Transdecoder v.5.7.0 to predict Open Reading Frames (ORFs). Then unigenes were queries against the SwissProt database (release 2023_02) using blast^28^, Pfam database (release 35.0) using HMMER^29^, and Rfam (release 14.9) using infernal v1.1.4^30^. The annotations were associated with Gene Ontology (GO) terms from SwissProt and Pfam databases. In addition, Trinotate was used to predict transmembrane regions (tmHMM v2.0c43^31^) and signal peptide cleavage sites (signalP v6^32^). The results of these analyses were loaded into a local SQLite database and merged using Trinotate.

### Statistics

R v4.3.0 with the library TrinotateR (https://github.com/cstubben/trinotateR) was used for summarisation and visualizations of the obtained transcriptome assembly. For better clarity of the results, GO terms in the figures were filtered with cut-off of 1000 genes (terms with less than 1,000 genes are not included).

## Data Records

The filtered and cleaned original RNA sequencing data have been deposited at the NCBI Sequence Read Archive under the SRA study accession SRX20705925-SRX20705930^33^. This Transcriptome Shotgun Assembly project has been deposited at DDBJ/EMBL/GenBank under the accession GKNA00000000^34^. The version described in this paper is the first version, GKNA01000000. A full functional annotation of the Trinity transcriptome assembly file of the assembly, including the contaminants (39,641 sequences) flagged by NCBI (https://github.com/ncbi/fcs) as not belonging to *N. praecox* are available as a supplementary.tsv file at Zenodo as well as the list of genes, their counts and transcripts per million (TMP) values (10.5281/zenodo.10148119)^35^.

## Technical Validation

### Quality of the raw reads and assembly validation

**Fig. 2:**
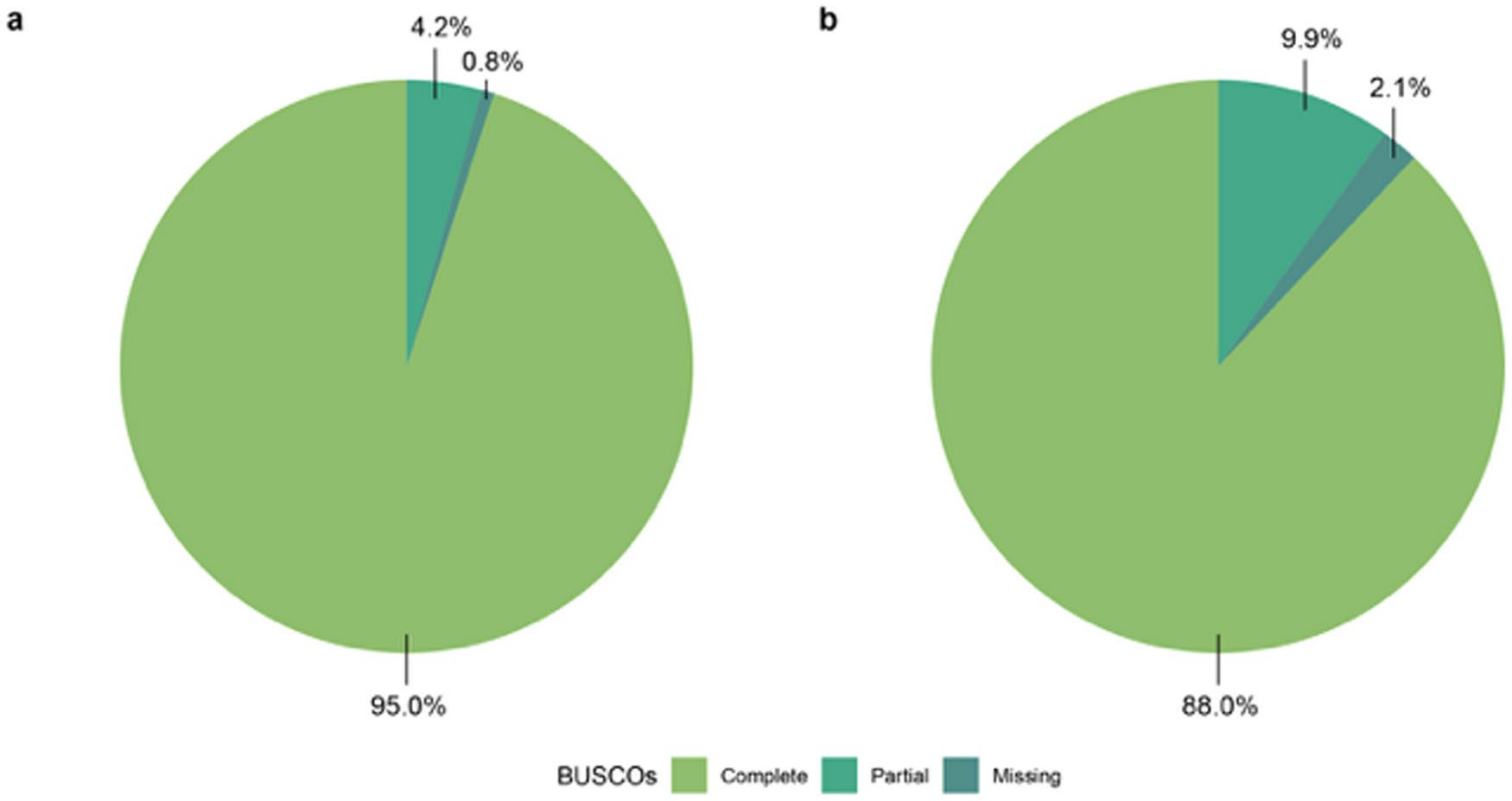
BUSCO transcriptome assessment of (**a**) all transcripts and (**b**) protein-coding genes.

Over 800 million 150 bp preprocessed reads were obtained from six biological samples of *N. praecox*. After trimming, filtering, and error correction, approximately 689 million (86% of raw reads) of high-quality paired reads were retained and used for *de novo* assembly. The initial Trinity assembly yielded 210,927 transcripts with an N50 of 1,343 bp. BUSCO score for the initial assembly against orthologs from Viridiplantae showed 95.1% complete, 4.2% fragmented, and 0.7% missing genes. Reducing the redundancy of the initial assembly resulted in an assembly of 177,907 transcripts with an N50 of 1,154 bp, an average sequence length of 834 bp, and a GC content of 44.0%. The assembly showed a reads mapped back to the transcriptome (RMBT) value of 62.3%, whereas transcriptome BUSCO completeness scores for the final assembly showed that the final assembly was 95.0% complete and 4.2% fragmented (Fig. 2a). The final assembly exhibited low levels of missing single-copy orthologs (0.8% missing), indicating good coverage and quality of the assembly. On the other hand, the BUSCO completeness score for protein-coding genes showed the final assembly was 88.0% complete and 9.9% fragmented (Fig. 2b). The percentage of missing single-copy orthologs was higher compared to the BUSCO score for all transcripts (2.1%). Additionally, ExN50 for all transcripts was calculated as it has been suggested to be more informative than the contig N50 and, therefore, a more reliable measure of transcriptome assembly quality^36^. Our assembly showed a peak saturation point at 90% of the normalized expression data.

**Fig. 3:**
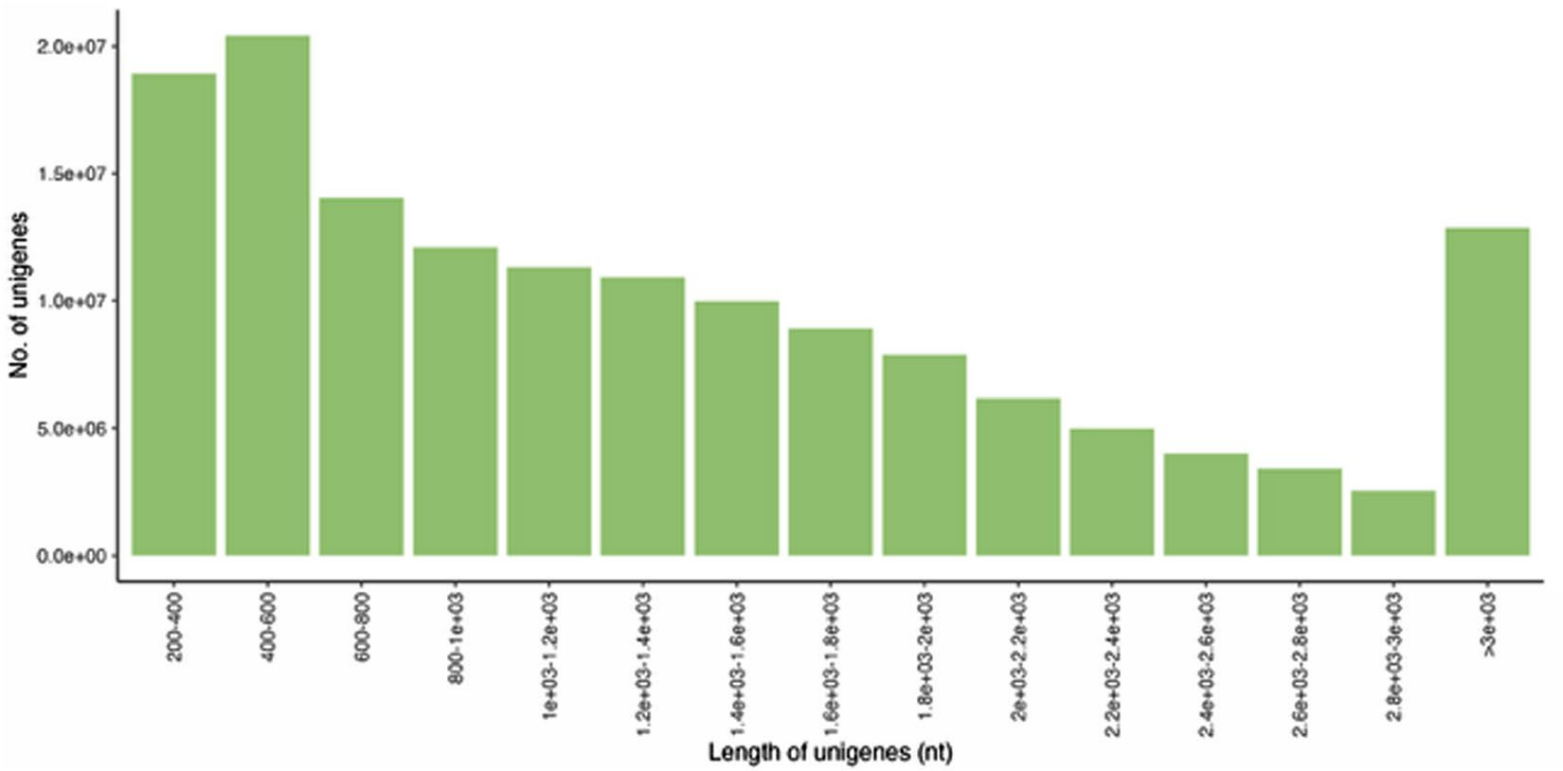
Length distribution for unigenes after reduced redundancy in the assembly.

After reducing redundancy, the length distribution of unigenes was assessed. Most unigenes were 400–600 nucleotides long, and their number decreased with the increasing length. An increased number of unigenes is then detected at the length of >3000 nucleotides (Fig. 3). Differential expression analysis yielded 11,128 differentially expressed genes: between plants grown on the mental-enriched site Žerjav, and the control location in Lokovec. 5,074 genes were down-regulated and 6,054 up-regulated at the metal-enriched site in Žerjav. If contigs were filtered by annotation and only those an annotation for plant taxa were included in the analysis, 3,288 differentially expressed genes were observed. Of those 1,440 were up-regulated in plants at Žerjav and 1,848 were down-regulated at Žerjav, the metal-enriched site.

### Quality control of annotation

The quality of functional annotation depends on the read quality and on the reference data used in the analysis. Therefore, it is crucial to choose appropriate data source to achieve appropriate annotation quality. Search against the SwissProt database yielded results for 80,717 (59.65% of all) unigenes (Table 2), whereas the search for protein sequences found 53,142 (39.27% of all) and 51,479 (38.04% of all) matches for SwissProt and Pfam databases, respectively. Furthermore, 77,738 (57.45%) of unigenes were identified to possess trans-membrane regions and 3,639 (2.69%) were flagged for signal peptides. A search against Rfam identified 893 (0.61%) transcripts as belonging to non-mRNA families.

**Table 2:** The number of unique and total unigenes with annotation using Trinotate for the final *Noccaea praecox* transcriptome assembly (reduced redundancy).

	Unique	Total	% of total unique	% of total
Transcripts	135,323	135,323	100.00	100.00
BLASTx hits	80,593	80,717	59.56	59.65
BLASTx GO	16,146	78,105	11.93	57.72
Proteins	77,738	77,738	57.45	57.45
Transmembrane regions	1,455	77,738	1.08	57.45
KEGG	19,228	76,114	14.21	56.25
BLASTp hits	49,024	53,142	36.23	39.27
BLASTp GO	14,634	51,816	35.18	38.29
Pfam hits	47,612	51,479	10.81	38.04
Pfam GO	1,897	31,069	1.40	22.96
Signal peptides	2,426	3,639	1.79	2.69
Rfam hits	675	893	0.50	0.61
eggnog	249	831	0.18	0.66

**Fig. 4:**
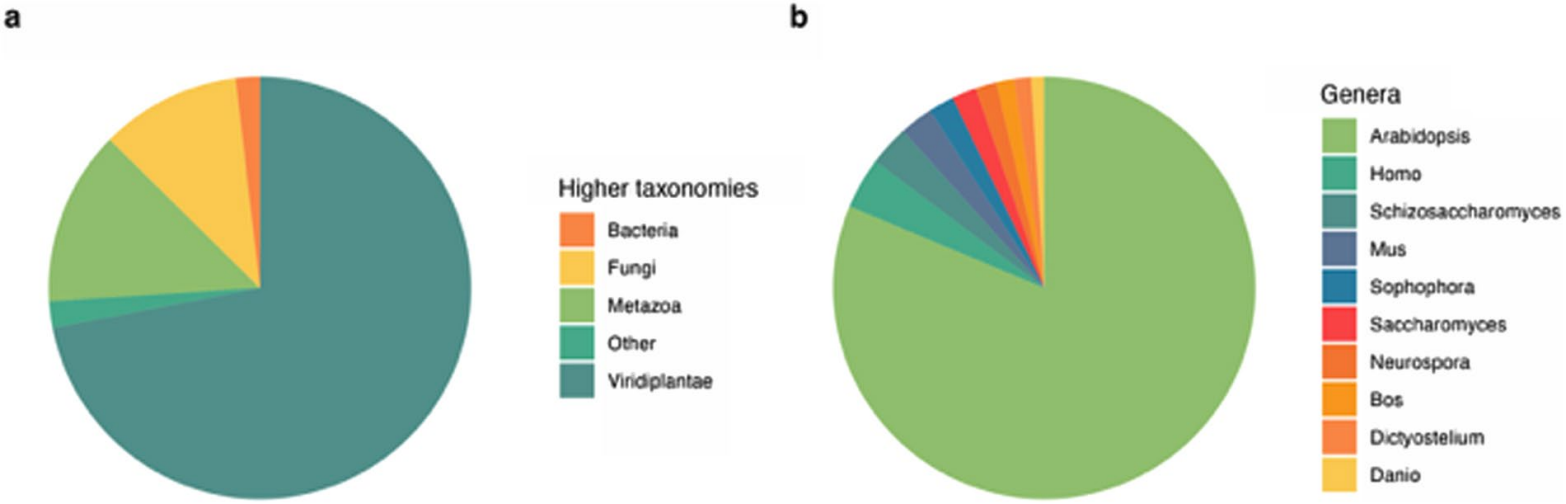
BLASTp taxonomic results against SwissProt database: (**a**) at higher taxonomic levels and (**b**) at the genus level.

The highest number of searches for proteins against SwissProt showed affinity to Viridiplantae (Fig. 4a) with 44,264 transcripts (83.3% of all hits), followed by Metazoa and Fungi. At the genus level, the highest number of hits against the database was assigned to Arabidopsis, with 42,462 transcripts (54.6% of all hits) (Fig. 4b).

**Fig. 5:**
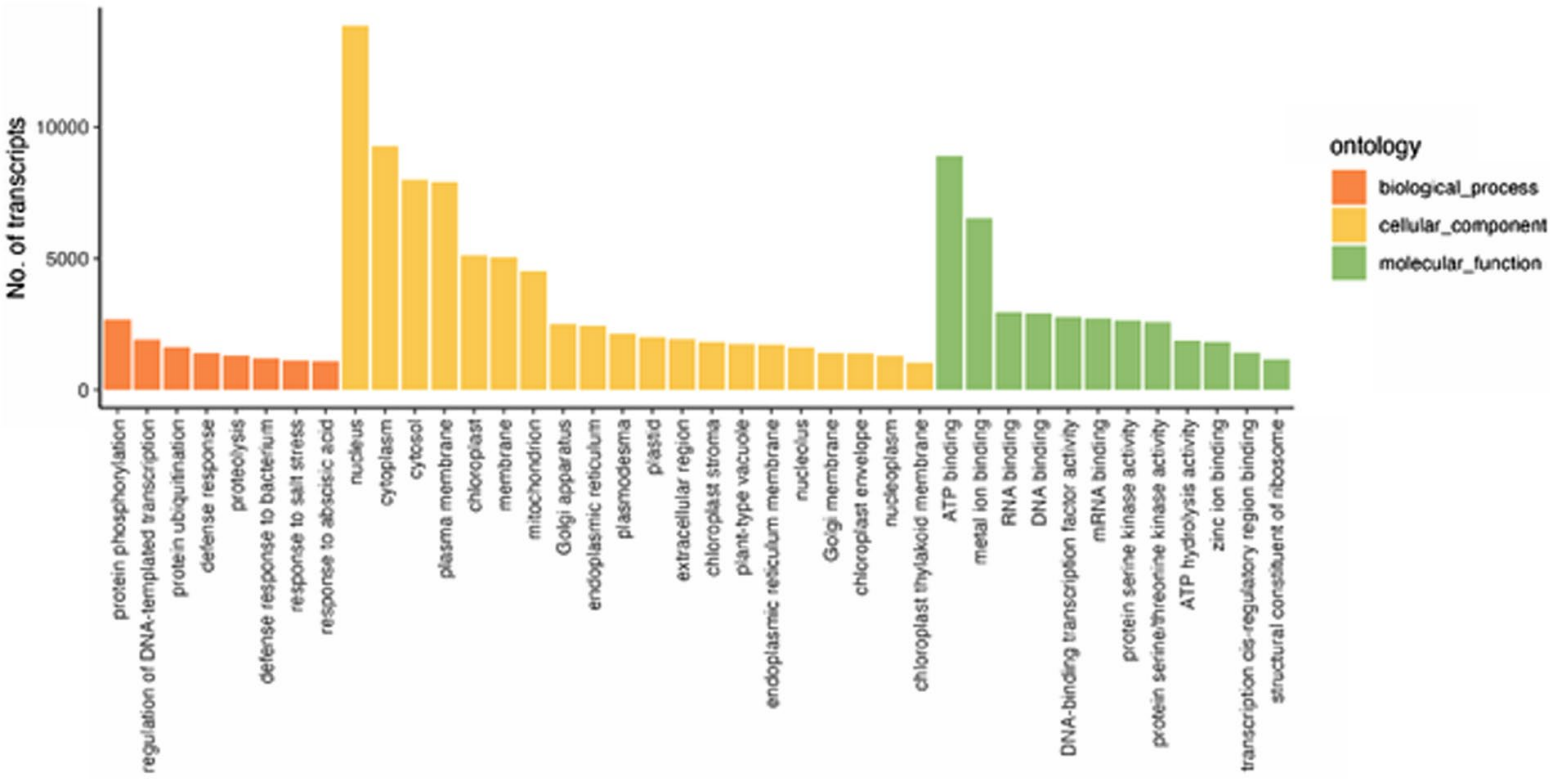
Distribution of Gene Ontology (GO) classified into three functional categories: biological process, cellular component, and molecular function. GO categories with less than 1,000 unigenes were removed for this representation.

We then classified the transcripts based on their annotated GO terms (Fig. 5). In the Biological processes category (45,254, 32.2% of all transcripts with GO term annotation), the three top GO terms are ‘protein phosphorylation’ (2,666, 1.7%), ‘regulation of DNA-templated transcription’ (1,912, 1.2%) and ‘defense response’ (1,632, 1.1%). Cellular Component category has 46,400 (34.1%) transcripts with GO term annotation, among which ‘nucleus’ (13,850, 11.5%), ‘cytoplasm’ (9,273, 7,7%), ‘cytosol’ (7,994, 6.7%), and ‘plasma membrane’ (7,904, 6.6%) are the most abundant. There are 44,547 (32.7%) transcripts identified within the Molecular Function category, with ‘ATP binding’ and ‘metal ion binding’ having the largest number of matched transcripts, with 8,898 (7.6%) and 6,526 (5.6%), respectively.

**Fig. 6:**
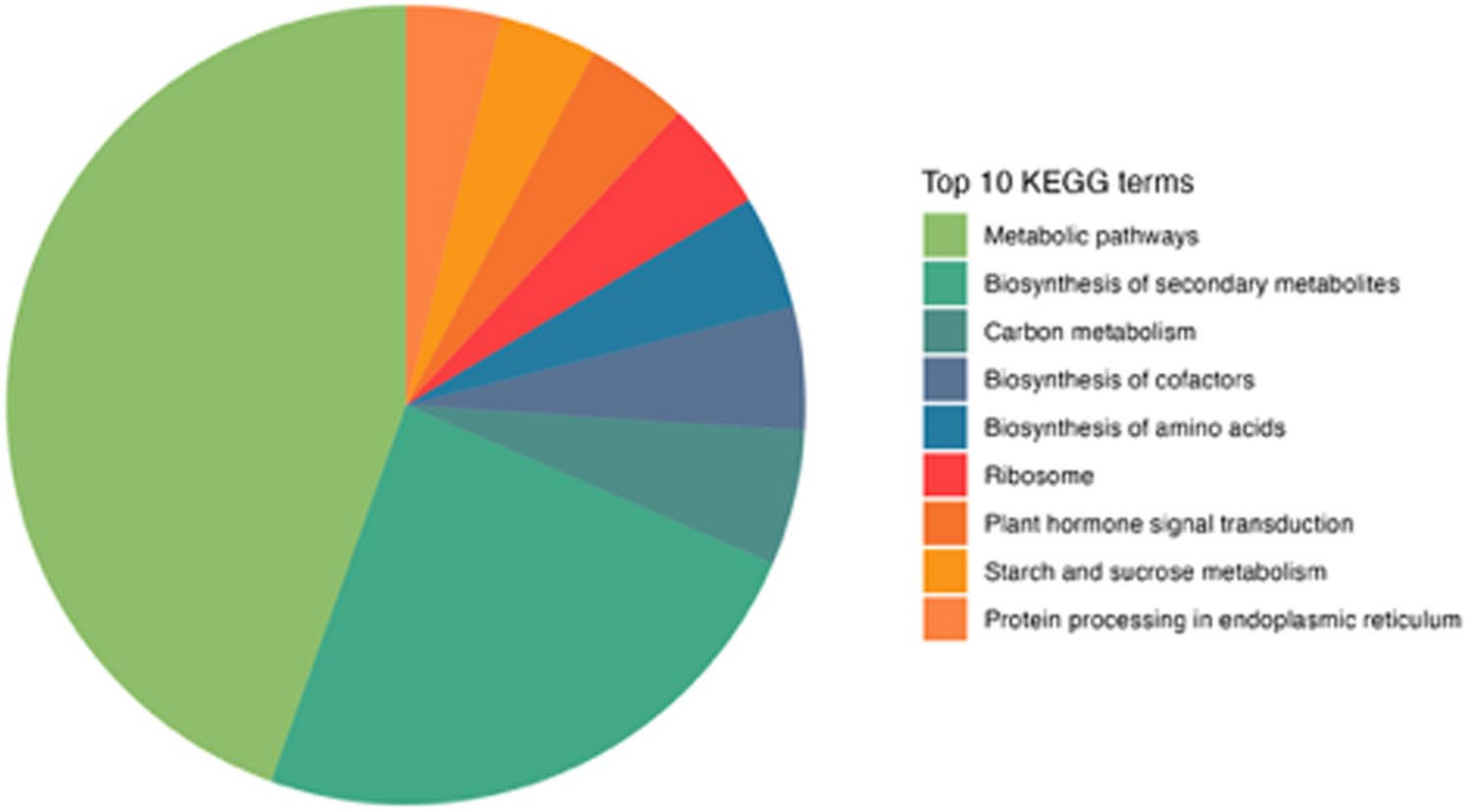
Annotations against the KEGG database for A. thaliana genes. The top ten KEGG pathways with the highest number of unigenes are represented.

In the end, we examined the annotations from the KEGG database for the *A. thaliana* transcripts (Fig. 6). The largest number of transcripts was annotated within ‘Metabolic pathways’ (9,176, 44.5% of all transcripts with KEGG annotation) and ‘Biosynthesis of secondary metabolites’ (4,947, 24.0% of all transcripts with KEGG annotation).

## Data Availability

The specific codes for analyses of RNA-seq data are available at https://github.com/matevzl533/Noccaea_praecox_transcriptome.
